# Phytochemical Compositions and Biological Activities of Essential Oil from *Xanthium strumarium* L.

**DOI:** 10.3390/molecules20047034

**Published:** 2015-04-17

**Authors:** Javad Sharifi-Rad, Seyedeh Mahsan Hoseini-Alfatemi, Majid Sharifi-Rad, Mehdi Sharifi-Rad, Marcello Iriti, Marzieh Sharifi-Rad, Razieh Sharifi-Rad, Sara Raeisi

**Affiliations:** 1 Zabol Medicinal Plants Research Center, Zabol University of Medical Sciences, Zabol 61615-585, Iran; E-Mail: javad.sharifirad@gmail.com; 2 Department of Pharmacognosy, Faculty of Pharmacy, Zabol University of Medical Sciences, Zabol 61615-585, Iran; 3 Pediatric Infections Research Center, Mofid Children Hospital, Shahid Beheshti University of Medical Sciences, Tehran 15468-15514, Iran; E-Mail: m.hoseinialfatemi@gmail.com; 4 Department of Range and Watershed Management, Faculty of Natural Resources, University of Zabol, Zabol 98615-538, Iran; E-Mail: majid.sharifirad@gmail.com; 5 Zabol University of Medical Sciences, Zabol 61663335, Iran; E-Mail: mehdi_sharifirad@yahoo.com; 6 Department of Agricultural and Environmental Sciences, Milan State University, via G. Celoria 2, Milan 20133, Italy; 7 Department of Chemistry, Faculty of Science, University of Zabol, Zabol 98615-538, Iran; E-Mail: marzieh.sharifirad@gmail.com; 8 Department of Biology, Faculty of Science, University of Sistan and Baluchestan, Zahedan 33431063, Iran; E-Mail: razieh.sharifirad@gmail.com; 9 Department of Fishery, Gorgan University of Agricultural Sciences and Natural Resources, Gorgan 49138-15739, Iran; E-Mail: sarah.raeisi69@gmail.com

**Keywords:** cocklebur, isoprenoids, antimicrobial agents, antibacterial activity, antifungal activity, scolicidal activity

## Abstract

The chemical composition of the essential oil (EO) from fresh cocklebur (*Xanthium strumarium* L.) leaves was investigated by GC-MS. The antimicrobial activity of the EO was tested against Gram-positive and Gram-negative bacteria and fungi. Scolicidal activity was assayed against *Echinococcus granulosus* protoscolices. In total, 34 compounds were identified, accounting for 98.96% of the EO. The main compounds in the EO were *cis*-β-guaiene (34.2%), limonene (20.3%), borneol (11.6%), bornyl acetate (4.5%), β-cubebene (3.8%), sabinene (3.6%), phytol (3.1%), β-selinene (2.8%), camphene (2.2%), α-cubebene (2.4%), β-caryophyllene (1.9%), α-pinene (1.8%) and xanthinin (1.04%). The antibacterial and antifungal screening of the EO showed that all assayed concentrations significantly inhibited the growth of *Staphylococcus aureus*, *Bacillus subtilis*, *Klebsiella pneumoniae*, *Pseudomonas aeruginosa*, *Candida albicans* and *Aspergillus niger* (MIC = 0.5 ± 0.1, 1.3 ± 0.0, 4.8 ± 0.0, 20.5 ± 0.3, 55.2 ± 0.0 and 34.3 ± 0.0 µg/mL, respectively). The scolicidal assay indicated that the EO exhibited a significant activity against *E. granulosus* protoscolices. To the best of our knowledge, this is the first report on the scolicidal activity of *X. strumarium*. Because of the emergence of antimicrobial drug resistance, the study of new effective natural chemotherapeutic agents, such as the *X. strumarium* EO, possibly with low side effects, represents a very promising approach in biomedical research.

## 1. Introduction

For a long time, aromatic and medicinal plants have played an important role as (phyto) therapeutic agents of both pharmacological and economic relevance [1,2,3,4]. In developing countries, due to economic constraints, nearly 80% of the population still depends on plant extracts as a source of natural remedies. Noteworthily, the excessive and repeated use of pharmaceuticals in modern medicine has caused the selection of antibiotic resistant microbial strains, thus reducing the number of antibiotics available to treat clinical infections [5,6,7,8,9,10], therefore, the use of medicinal and aromatic plants as a source of new therapeutic agents continues to be a pivotal element in traditional health care systems [10]. In addition, phytochemicals from these plants may also serve as precursors or lead compounds for the development of new pharmaceuticals [3,11,12].

Cocklebur (*Xanthium strumarium* L.) is an annual plant species belonging to the Asteraceae family. In Iran, *X. strumarium* is available between August and September, where it competes with a number of agronomic crops. In many countries, different plant organs, especially fruits and roots, are used as remedies [13]. Extracts from these plant organs were found to possess antifungal [14], anti-inflammatory [15,16], antileishmanial [14], antitrypanosomal [17], hypoglycemic [18], anthelmintic [19], antiulcerogenic [20], diuretic [21] and anticancer [22] activities.

Essential oils are complex mixtures of lipophilic, volatile and aromatic plant secondary metabolites. The principal constitutes of essential oils include mono- and sesquiterpenes, arising from the isoprenoid pathway, and their oxygenated derivatives such as ketones, alcohols, aldehydes, esters, oxides and phenols [23]. Several studies have reported the biocide activity of essential oils against many different agents, including clinically relevance pathogens [24,25,26].

The most important chemical constituents of *X. strumarium* include phenolic compounds as thiazolidinediones, chlorogenic acids, ferulic acids [27], 1,3,5-tri-*O*-caffeoyl quinic acid, 1,5-di-*O*-caffeoyl quinic acid, caffeic acid [28], as well as isoprenoids such as strumasterol, β-sitosterol [29], monoterpene and sesquiterpene hydrocarbons [30], triterpenoid saponins [29] and xanthanolide sesquiterpene lactones [31]. Based on these premises, the main aim of the this study was to carry out *in vitro* assays to estimate the antimicrobial and scolicidal activities of essential oil extracted from leaves of *X. strumarium* grown in Iran.

## 2. Results and Discussion

### 2.1. Chemical Composition of X. strumarium Leaf Essential Oil

The chemical composition of essential oil extracted from the leaves of *X. strumarium* is shown in Table 1. 

**Table 1:** Phytochemical composition of *Xanthium strumarium* L. leaf essential oil.

No.	Name of Compound	RI *	Relative % in Essential Oil
1	α-Pinene	939	1.8
2	Camphene	953	2.2
3	Sabinene	976	3.6
4	Myrcene	991	0.5
5	*p*-Cymene	1026	t
6	Limonene	1032	20.3
7	Linalool	1099	0.9
8	*trans*-Verbenol	1135	0.4
9	Borneol	1166	11.6
10	*trans*-Carveol	1217	0.9
11	Bornyl acetate	1286	4.5
12	Tridecane	1299	0.2
13	α-Cubebene	1351	2.4
14	Eugenol	1356	t
15	α-Ylangene	1373	t
16	α-Copaene	1376	0.2
17	β-Cubebene	1390	3.8
18	β-Elemene	1391	0.2
19	β-Caryophyllene	1418	1.9
20	β-Gurjunene	1432	0.4
21	α-Humulene	1454	0.6
22	Germacrene D	1480	t
23	β-Selinene	1485	2.8
24	*cis*-β-Guaiene	1490	34.2
25	Valencene	1491	0.4
26	α-Muurolene	1499	t
27	γ-Cadinene	1513	0.1
28	Cubebol	1514	0.2
29	δ-Cadinene	1525	0.2
30	Xanthatin	1575	t
31	α-Cadinol	1613	t
32	*epi*-α-Cadinol	1654	0.4
33	Phytol	1821	3.1
34	Xanthinin	2341	1.0
	Monoterpene hydrocarbons		28.8
	Oxygenated monoterpenes		17.9
	Sesquiterpene hydrocarbons		47.2
	Oxygenated sesquiterpenes		0.6
	Others		4.3
	Total identified		98.9

* RI: retention index; t: traces, concentration less than 0.05%.

GC-MS analysis revealed that the main components of the essential oil were *cis*-β-guaiene (34.2%), limonene (20.3%), borneol (11.6%), bornyl acetate (4.5%), β-cubebene (3.8%), sabinene (3.6%), phytol (3.1%), β-selinene (2.8%), camphene (2.2%) α-cubebene (2.4%), β-caryophyllene (1.9%), α-pinene (1.8%) and xanthinin (1.04%). Scherer *et al*. [32] studied the *X. strumarium* leaf essential oil from São Paulo, Brazil: among the 24 components identified in that work, β-guaiene was the most abundant (79.6%). Esmaeili *et al*. [33] collected *X. strumarium* plants at full flowering stage, from Khoramabad, Lurestan Province, Iran, and obtained the essential oil from stems and leaves. They reported that 22 compounds (86.4%) were identified in the stem essential oil, among which bornyl acetate (19.5%), limonene (15.0%) and β-selinene (10.1%) were the most abundant. In the leaf essential oil, 28 components were identified (85.2%), characterized by higher amounts of limonene (24.7%) and borneol (10.6%). Our results are in agreement with previous studies: no significant qualitative difference was observed in the essential oil composition, whereas any quantitative differences may be due to genetic, environmental and ecological factors.

### 2.2. Antibacterial, Antifungal and Scolicidal Activities

The antibacterial and antifungal activity results are summarized in Table 2 and Table 3, respectively. *X. strumarium* essential oil significantly inhibited the growth of Gram-positive (*S. aureus* and *B. subtilis*) and Gram-negative (*K. pneumoniae*) bacteria (*p* < 0.05). MIC for *S. aureus*, *B. subtilis* and *K. pneumoniae* were 0.5 ± 0.1, 1.3 ± 0.0 and 4.8 ± 0.0 µg/mL of essential oil, respectively. *S. aureus* was the most sensitive microorganism, because of its very low MIC. *P. aeruginosa* was slightly inhibited in the disc diffusion assay, and its MIC was 20.5 ± 0.3 μg/mL of essential oil in the broth dilution assay. In addition, the essential oil significantly inhibited *C. albicans* and *A. niger* (*p* < 0.05), at all the assayed concentrations. MIC for *C. albicans* and *A. niger* were 55.2 ± 0.0 and 34.3 ± 0.0 µg/mL of essential oil, respectively.

The mortality rates of *E. granulosus* protoscolices after treatment with different concentrations of *X. strumarium* leaf essential oil are reported in Table 4. As exposure time and essential oil concentration increased, percentage mortality rised. Therefore, exposure to the essential oil for 60 min, at 2.5, 5, 10 and 20 mg/mL resulted in 58.7%, 64.48%, 68.48% and 79.22% inhibition, respectively. After 60 min, the mortality in the control was 43.56%.

**Table 2:** Antibacterial activity of *Xanthium strumarium* L. leaf essential oil against gram-positive and gram-negative bacterial strains.

Essential Oil (µg/mL)	*Staphylococcus aureus*	*Bacillus subtilis*	*Klebsiella pneumoniae*	*Pseudomonas aeruginosa*
10	42.5 ± 0.1 e ^§^	22.3 ± 0.0 e	20.31 ± 0.2 d	14.5 ± 0.0 d
20	52.7 ± 0.0 d	36.31 ± 0.2 d	22.8 ± 0.0 d	42.22 ± 0.1 c
40	77.7 ± 0.0 c	47.22 ± 0.3 c	44.2 ± 0.0 c	43.33 ± 0.0 c
60	77.9 ± 0.1 c	49.22 ± 0.0 c	53.6 ± 0.0 b	53.4 ± 0.0 b
80	89.33 ± 0.0 b	80.39 ± 0.5 b	57.4 ± 0.2 a	62.8 ± 0.1 a
100	124.42 ± 0.0 a	98.5 ± 0.0 a	58.1 ± 0.0 a	64.4 ± 0.0 a
DMSO *	2.2 ± 0.0 f	2.21 ± 0.0 g	3.2 ± 0.0 f	2.2 ± 0.0 f
Ampicillin	17.5 ± 0.0 g	15.8 ± 0.0 f	-	-
Gentamicin	-	-	10.3 ± 0.0 e	10.2 ± 0.0 e
MIC	0.5 ± 0.1	1.3 ± 0.0	4.8 ± 0.0	20.5 ± 0.3

^§^ Data are expressed as mean ± SD of inhibition zone diameter (mm) for different concentrations of essential oil, controls and minimum inhibitory concentration (MIC) (µg/mL); the values with different letters within a column are significantly different (*p* < 0.05; LSD); * DMSO: dimethyl sulfoxide.

**Table 3:** Antifungal activity of *Xanthium strumarium* L. leaf essential oil against fungal strains.

Essential Oil (µg/mL)	*Candida albicans*	*Aspergillus niger*
10	3.2 ± 0.0 g ^§^	2.3 ± 0.0 e
20	9.5 ± 0.0 f	2.5 ± 0.2 e
40	15.9 ± 0.2 d	11.2 ± 0.0 c
60	29.2 ± 0.0 c	23.5 ± 0.1 b
80	36.7 ± 0.3 b	23.9 ± 0.1 b
100	44.1 ± 0.3 a	35.2 ± 0.5 a
DMSO *	3.2 ± 0.1 g	2.1 ± 0.0 e
Ketoconazole	11.5 ± 0.0 e	10.3 ± 0.0 d
MIC	55.2 ± 0.0	34.3 ± 0.0

^§^ Data are expressed as mean ± SD of inhibition zone diameter (mm) for different concentrations of essential oil, controls and minimum inhibitory concentration (MIC) (µg/mL); the values with different letters within a column are significantly different (*p* < 0.05; LSD); * DMSO: dimethyl sulfoxide.

According to Scherer *et al*. [32], leaves of *X. strumarium* exhibited powerful antimicrobial activity against *S. aureus*, *Escherichia coli*, *Salmonella thyphimurium*, *P. aeruginosa* and *Clostridium perfringens.* In addition, they showed that *S. aureus* was the most susceptible microorganism followed by *E. coli* and *P. aeruginosa*, while *S. typhimurium* and *C. perfringens* were the most resistant to the *X. strumarium* essential oil. Rad *et al*. [13] investigated the antibacterial activity of *X. strumarium* on methicillin-susceptible (MSSA) and methicillin-resistant *S. aureus* (MRSA), showing that the plant extracts were effective on both strains, though their antibacterial activity was higher on the MSSA one. Similarly, Jawad *et al.* [34] reported that *X. strumarium* extract exhibited antimicrobial activity against *S. aureus*, *B. subtilis*, *Proteus vulgaris*, *Candida pseudotropicalis* and *C. albicans*. Gautam *et al*. [35] investigated *X. strumarium* extracts for *in vitro* antimycobacterium activity, and found that the ethylacetate and MeOH-petroleum ether extracts were effective against *Mycobacterium smegmatis* and *M. tuberculosis*. Amerjothy *et al*. [36] studied the hexane, alcoholic and ethylacetate extracts of *Xanthium indicum* Koen leaves for their antimicrobial activity. Hexane extract showed significant inhibition against *P. aeruginosa, S. aureus, Aspergillus niger* and *C. albicans*; ethylacetate extract inhibited *S. aureus, A. niger* and *E. coli*; alcoholic extract was active only against *S. aureus*. Antifungal activity of *X. strumarium* was also documented against both pathogenic and non-pathogenic fungi by Bisht and Singh [37], due to the presence of terpenes, limonene and carveol.

**Table 4:** Scolicidal activity of *Xanthium strumarium* leaf essential oil against *Echinococcus granulosus*.

Concentration(mg/mL)	Exposure Time(min)	Protoscolices	Dead Protoscolices	Mortality (%)
2.5	10	1150.01± 33.00 ^§^	354.33 ± 45.22	30.78
	20	1322.17 ± 42.11	432.77 ± 22.15	32.73
	30	1432.04 ± 73.00	666.05 ± 62.00	46.51
	60	1153.22 ± 76.12	677.00 ± 11.00	58.7
	Control *	1245.00	542.35	43.56
5	10	1270.11 ± 39.8	458.00 ± 45.00	36.06
	20	1125.11 ± 44.32	488.01 ± 56.22	43.37
	30	989.28 ± 34.34	434.00 ± 66.00	43.86
	60	1377.00 ± 24.11	888.00 ± 11.00	64.48
	Control	1245.0	542.35	43.56
10	10	1444.34± 12.21	589.44 ± 56.12	40.81
	20	1334.55 ± 71.22	612.44 ± 19.29	45.89
	30	1254.99 ± 33.31	746.69 ± 36.17	59.49
	60	1394.72 ± 61.22	955.19 ± 23.7	68.48
	Control	1245.00	542.35	43.56
20	10	1149.49 ± 11.41	589.47 ± 17.11	51.28
	20	1393.39 ± 14.2	757.33 ± 49.11	54.35
	30	844.56 ± 42.12	588.82 ± 42.72	66.16
	60	977.22 ± 19.12	774.18 ± 12.9	79.22
	Control	1245.00	542.35	43.56

^§^ Values are mean ± SD of three replicates; * in the control, protoscolices were treated only with saline + Tween-80 solution.

Among the most representative constituents found in our essential oil, the sesquiterpene β-caryophyllene was extensively investigated because of its several biological activities, including antimicrobial [38,39], insecticidal [40,41], anti-inflammatory [42,43], anticarcinogenic [44,45,46,47,48] and local anaesthetic [49] activities.

Similarly, many studies showed the antimicrobial activity of α-pinene and eugenol on Gram-positive bacterial strains (*S. aureus*, *Streptococcus pyogenes*, *S. epidermidis* and *Streptococcus pneumoniae*) and fungi (*Cryptococcus neoformans* and *C. albicans*) [23,50,51]. In our study, both α-pinene (1.8%) and eugenol (trace amount) were detected in *X. strumarium* essential oil, as well as limonene (20.3%) and linalool (0.9%) (Table 1) [52]. Aggarwal *et al.* [53] reported that limonene was particularly efficient in inhibiting the proliferation of a variety of microorganisms that cause food spoilage. Özek *et al.* [54] demonstrated that linalool enantiomers possessed the same antimicrobial activity against several microorganisms, specifically against the protozoan *Plasmodium falciparum* and the fungus *Botrytis cinerea*.

Mulyaningsih *et al.* [55] studied antibacterial activity of *Kadsuralongi pedunculata* essential oil and its major constituents against MRSA and vancomycin-resistant *Enterococcus faecalis*. Fifty compounds were identified, including δ-cadinene (21.79%), camphene (7.27%), borneol (6.05%), cubenol (5.12%) and δ-cadinol (5.11%), and the authors reported that camphene and borneol exhibited antimicrobial activity. Borneol (11.6%), camphene (2.2%), δ-cadinene (0.2%) and α-cadinol (trace amount) were found in our *X. strumarium* essential oil (Table 1). δ-Cadinene inhibited the growth of *Propionibacterium acnes* and *S. mutans* [56]. Pérez-Lopez *et al.* [57] essayed the essential oil obtained from the fruit of *Schinus molle* against *S. pneumonia* resistant to antibiotics, and identified δ-cadinene as the principal active ingredient.

Xanthinin (1.04%) was found in *X. strumarium* essential oil (Table 1). This compound was previously isolated from the extracts of *X. spinosum* and was active against *Colletotrichum gloesporoides*, *Trichothecium roseum*, *Bacillus cereus* and *Staphylococcus aureus* [58]. Little *et al*. [59] reported that alcoholic extract of xanthinin in concentration of 0.01%–0.1% showed high antimicrobial activity against fungi and gram-negative bacteria.

Inoue *et al.* [60] examined the bactericidal activity of three diterpenes, i.e. phytol, terpenone and geranylgeraniol, showing that these compounds were effective against *S. aureus*. Similarly, Pejin *et al*. [61] investigated the antimicrobial activity of phytol against eight bacterial and eight fungal strains. It was proven phytol to be active against all tested bacteria and fungi. The amount of phytol in *X. strumarium* essential oils was 3.1% (Table 1).

Maggiore *et al*. [62] reported the efficacy of *Thymus vulgaris* and *Origanum vulgare* essential oils and thymol on *E. granulosus* protoscoleces and cysts [63]. Mahmoudvand *et al*. [64] studied scolicidal activity of black cumin seed (*Nigella sativa*) essential oil on hydatid cysts, and thymoquinone, *p*-cymene, carvacrol and longifolene were found to be the main components of the essential oil. To the best of our knowledge, this is the first report on the scolicidal activity of *X. strumarium*.

## 3. Experimental Section 

### 3.1. Plant Material

The *Xanthium strumarium* L. leaves were collected between August-September 2013 from area of Hamun Lake of Zabol (31°1'43'' N, 61°30'4'' E), Sistan and Baluchestan Province, Iran. The plant was taxonomically identified at the Department of Botany of Shahid Beheshti University of Medical Sciences, Tehran, Iran, where a voucher specimen was conserved.

### 3.2. Essential Oils Extraction

Fresh leaves (1 kg) were detached from the stem and dried in the shade for 96 h. Then, they were chopped and hydro-distilled for 3 h utilizing an all-glass Clevenger-type apparatus. The distillate was saturated with sodium chloride (NaCl) (Merck, Darmstadt, Germany) and the oil was extracted with *n*-hexane (Merck) and dichloromethane (Merck). The essential oil obtained was dried over anhydrous sodium sulphate (Sigma-Aldrich, St. Louis, MO, USA) and stored at 4 °C before gas chromatography coupled to mass spectrometry (GC-MS) analysis and bioassays.

### 3.3. Identification of Essential Oil Constituents

The leaf essential oil was analyzed by GC-MS. A Shimadzu 17A gas chromatograph coupled with a Shimadzu QP-5000 quadrupole mass spectrometer and Varian 3800 gas chromatograph coupled with FID detector was used. The extracted compounds were separated on DB-5 fused silica capillary column (30 m × 0.25 mm × 0.25 µm film thickness). Helium was used as carrier gas with a 1.0 mL/min flow rate. The analyses were carried out by a splitless injection (1 µL), with the injector set at 230 °C. The oven temperature program used was 60–240 °C at 3 °C /min and the final temperature was held for 8 min. The GC/MS interface and FID detector were sustained at 240 °C and 250 °C, respectively. Retention indices for all constituents were determined based on the method using *n*-alkanes as standard. Retention indices were determined using retention times of *n*-alkanes that were injected after the essential oil under the same chromatographic conditions. All data were acquired by collecting the full-scan mass spectra within the scan range 50–550 amu. Compounds were recognized using comparison of their mass spectra with the Wiley GC-MS Library and Adams Library [65,66].

### 3.4. Microbial Isolates, Antibacterial and Antifungal Activities

All microorganisms were obtained from the Persian Type Culture Collection (PTCC), Tehran, Iran. The essential oil was tested against three gram-negative bacteria: *Klebsiella pneumoniae* PTCC 1053 (American Type Culture Collection ATCC 10031), *Escherichia coli* PTCC 1330 (ATCC 8739) and *Pseudomonas aeruginosa* PTCC 1074 (ATCC 9027); three gram-positive bacteria *Staphylococcus aureus* PTCC 1112 (ATCC 6538), *Staphylococcus epidermis* PTCC 1114 (ATCC 12228) and *Bacillus subtilis* PTCC 1023 (ATCC 6633); and two fungi: *Aspergillus niger* PTCC 5010 (ATCC 9142) and *Candida albicans* PTCC 5027 (ATCC 10231).

Different concentrations of essential oil were evaluated against bacteria and fungi by disc diffusion method [67]. In brief, microorganisms were cultured at 37 °C for 14–24 h and the densities were adjusted to 0.5 McFarland standards at A_530_ nm (10^8^ CFU/mL). Then, 100 µL of the microbial suspensions (10^8^ CFU/mL) were spread on nutrient agar (Merck) plates (100 mm × 15 mm). The discs (6 mm diameter) were separately impregnated with 10 µL of different concentrations of essential oil (10, 20, 40, 60, 80 and 100 µg/mL) and placed on the inoculated agar. All the inoculated plates were incubated at 37 °C for 24 h. Ketoconazole (10 mg/disc), ampicillin (10 mg/disc) and gentamicin (10 mg/disc) were used as positive controls for fungi, gram-positive and gram-negative bacteria, respectively. Dimethyl sulfoxide (DMSO) was used as negative control. Antibacterial and antifungal activities were determined by measuring the zone of inhibition (mm). Minimal inhibitory concentration (MIC) values of the of essential oil versus each investigated microbial strain were determined by the microdilution assay in 96 multi-well microtiter plates, according to the standard procedure of the Clinical and Laboratory Standards Institute [68]. The bacterial and fungal strains were suspended in Luria-Bertani media and the densities were adjusted to 0.5 McFarland standard at 570 nm (10^8^ CFU/mL). Essential oil was dissolved in 50% DMSO to a final concentration of 10 mL. Each strain was assayed with samples that were serially diluted in broth to obtain concentrations ranging from 512.0 to 0.06 µg/mL. Overnight broth cultures of each strain were prepared and the final microorganism concentration in each well was adapted to 10^6^ CFU/mL. The optimal incubation conditions were 37 °C for 24 h. Medium without bacteria and fungi was the sterility control, whereas medium with bacteria and fungi, but without essential oil, was the growth control. The growth of bacteria and fungi was compared with that of the controls. The MIC values were visually detected and defined as the lowest essential oil concentrations with >95% growth inhibitory activity to the assessed microorganisms.

### 3.5. Scolicidal Activity

The *Echinococcus granulosus* protoscolices were obtained from the infected livers of calves killed in an abattoir used to study scolicidal activity. Animals were ethically treated according to the Helsinki Declaration. In this assay, hydatid fluid was collected together with protoscolices using the Smyth and Barrett method [69]. Briefly, hydatid fluid was conveyed to a glass cylinder. Protoscolices, settled at the bottom of the cylinder after 40 min, were washed 3 times with normal saline and their viability was confirmed by motility under a light microscope (Nikon Eclipse E200, Tokyo, Japan). Protoscolices were transferred into a dark receptacle containing normal saline and stored at 4 °C. Four concentrations of essential oil (2.5, 5, 10 and 20 mg/mL) were tested for 10, 20, 30 and 60 min. To prepare these concentrations, 25, 50, 100 and 200 µL of essential oil, added to test tubes, were dissolved in 9.7 mL of normal saline supplemented with 0.5 mL of Tween-80 (Merck) under continuous stirring. For each test, one drop of protoscolices-rich solution was added to 3 mL of essential oil solution, mixed slowly, and incubated at 37 °C. After each incubation period (10, 20, 30 and 60 min), the upper phase was gently removed so as not to disturb the protoscolices; then, 1 mL of 0.1% eosin stain was added to the remaining colonized protoscolices and mixed slowly. The supernatant was discarded after incubating for 20 min at 25 °C. The remaining pellet of protoscolices (no centrifugation performed) was smeared on a manually scaled glass slide, covered with a cover glass, and evaluated under a light microscope. The percentage of dead protoscolices was determined after counting a minimum of 600 protoscolices. In the control, protoscolices were treated only with normal saline + Tween-80.

### 3.6. Statistical Analysis

Essential oil was extracted and tested in triplicate for chemical analysis and bioassays. Data were subjected to analysis of variance (ANOVA) following an entirely randomized design to determine the least significant difference (LSD) at *p* < 0.05, using statistical software package (SPSS v. 11.5, IBM Corporation, Armonk, NY, USA). All results are expressed as mean ± SD.

## 4. Conclusions

Our results indicated *X. strumarium* as a promising source on antimicrobial agents, with potential in biomedical applications. However, *in vivo* studies on this medicinal plant are needed to determine pharmacokinetics and toxicity of the active components and their side effects. In addition, the antimicrobial, antifungal and scolicidal activities may be increased by purifying active constitutes and determining proper dosages for effective therapies. This would avoid the prescription of inappropriate treatments, a usual practice among many traditional herbal practitioners. Finally, a particular application of *X. strumarium* plant may involve the field of food hygiene, to reduce the risk of food contamination and to control the food-borne diseases.
